# The effects of family relationships on vocational college students’ mental health

**DOI:** 10.3389/fpsyg.2026.1711541

**Published:** 2026-02-12

**Authors:** Yunpeng Du, Jiayue Ma, Mengying Fu, Mengyin Yan

**Affiliations:** Student Affairs Office, Shaanxi Vocational and Technical College, Xi’an, Shaanxi Province, China

**Keywords:** college students, emotion regulation, family relationships, mental health, questionnaire survey

## Abstract

**Introduction:**

Under conditions of normalized pandemic prevention, family relationships have significantly impacted vocational students’ mental health. However, the underlying emotional regulation mechanisms remain unclear. Therefore, this study investigated how emotion regulation and self-efficacy mediate the impact of family relationships on vocational students’ mental health.

**Methods:**

Using random sampling, we surveyed 2,026 students at a Chinese vocational college (November 2022), employing validated Chinese versions of the Emotion Regulation Questionnaire, Public Health Emergencies Questionnaire, Regulatory Emotional Self-efficacy Scale, and the Familial Aptitude and Cohesiveness Scale II.

**Results:**

All factors were significantly correlated (*p* < 0.05). Expressive suppression positively predicted mental health (*β* = 0.155, *t* = 4.787, *p* < 0.001), while self-efficacy for managing negative emotions negatively predicted mental health (*β* = −0.249, *t* = 7.446, *p* < 0.001). Family relationships induced mental health both directly and indirectly through emotion regulation.

**Discussion:**

Family relationships impact mental health via two pathways: directly and through emotion regulation mechanisms—particularly expressive suppression and the management of negative emotions. These findings highlight the mediating role of emotional regulation, offering evidence to support family-centered psychological interventions in vocational education settings.

## 1 Introduction

In the context of the COVID-19 pandemic, mental health should be further conceptualized as a dynamic and multidimensional construct shaped by prolonged stressors such as social isolation, uncertainty, disrupted routines, and increased academic and life pressures. Beyond the presence or absence of negative emotions (e.g., anxiety, depression, and loneliness), mental health during this period also reflects individuals’ psychological resilience, emotional regulation capacity, and ability to adapt to crisis-related changes. A person’s physical and mental growth, as well as completion of socialization, are greatly influenced by their family. Good family relationships significantly influence adolescents and college students. These relationships help adolescents and college students navigate transitional periods (Marth et al., 2022). Within the process of family systems influencing individual development, a secure and stable parent–child relationship serves as an important protective factor (Younas and Gutman, 2023; Zhu et al., 2015). One study (Zhu et al., 2023) suggested that family also influences an individual’s ability to adapt to society during socialization; for example, the family environment can predict adolescent behavior to a certain extent. The study by Gao et al. (2024) discovered a strong, favorable relationship between mental health and family cohesion and adaptation. According to the survey results of Zhang G. R. et al. (2023), Zhang L. et al. (2023), family cohesion, adaptability, and mental health are positively correlated. Due to their inability to obtain constructive emotional feedback or exchange with their families, children with lower levels of family cohesion and adaptation frequently experience unpleasant emotions, such as worry and loneliness. Conversely, in families with higher cohesion and adaptability, children’s levels of anxiety and loneliness are lower, and they can adjust their mindsets and flexibly cope with setbacks and challenges. Additionally, Lindert et al. (2025) suggested that family relationships with higher cohesion not only affect the mental health of family members but also effectively alleviate the effects of negative emotions, such as anxiety and depression, when family members experience them. A poor family atmosphere, characterized by low cohesion, tends to foster negative emotions such as irritability and loneliness (Hu and Tang, 2012). As the pandemic has confined adolescents and college students largely to the family environment, mental health can be understood as an adaptive psychological state emerging from the interaction between external stressors and internal coping resources, with family cohesion and adaptability playing a critical buffering and supportive role.

Emotional regulation is the process by which people manage the sources and expressions of emotions. Expressive suppression and cognitive reappraisal are the most widely used regulatory techniques. Regardless of whether emotion management affects mental or physical health, researchers continue to be interested in understanding its implications (Gross, 1998). Two categories of antecedent-focused and response-focused techniques can be distinguished based on the notion of emotion control (Gross and Muñoz, 1995). Regularly applying this approach to downregulate negative emotions, such as through cognitive reappraisal, can effectively reduce negative emotional experiences and the subsequent behavioral manifestations of these emotions. Expressive suppression is a response-based tactic that influences the behavioral manifestation of emotional reactions. Although suppression of negative emotions can lead to behavioral disclosure of such emotional displeasure, expressive suppression can also occur as an unintended understatement of positive emotions (Gross and John, 2003). Emotional regulation strategies can vary, with different outcomes regarding depressive emotions (Kökönyei et al., 2024). The selection of congruent emotion regulation strategies may help mitigate adverse emotions. This may enable individuals to utilize their competencies to deal with the consequences on their mental health.

An increased level of regulatory emotional self-efficacy is more related to a helping effect, which will assist in providing an individual with regulatory helpful power that will assist him or her in overcoming a poor mental state within the context of individuality; another study (McCraty et al., 1998) revealed a large number of researchers who have researched the topic. The associated research (Dou et al., 2013) suggests that they can optimize their sense of hope and control, positive self-concept, and life satisfaction by incorporating strategies to modify negative emotions or even exhibiting positive emotions. Furthermore, a person’s mental health is closely associated with their amount of regulatory emotional self-efficacy (Li and Quan, 2025). Based on a study on the regulation of emotional self-efficacy (Diener et al., 2010), mentally healthy adolescents can deploy mental resources in response to stress to an extent that increases positive feelings and reduces negative emotions.

A shortage of school research is evident because it fails to demonstrate the essence of family affiliation, emotion management, regulation of emotional self-sufficiency, and psychological health certification within the same shelter. A study on the implementation of family education among college students (Zhang et al., 2010) found that it has the potential to influence the emotional regulation and management among college students, particularly by enhancing regulatory self-efficacy related to emotions. Another study (Balogun, 2019) found that adolescents’ perception of family functioning influences their regulatory emotional self-efficacy and their ability to control negative emotions. Research on the process involving family relations, emotional regulation, regulatory emotional self-efficacy, and their coherence concerning the mental health of the vocational college population has not yet been conducted. According to the idea that the family is the perception of a person of subjective and objective support in the external environment, which implies more environmental variables, and according to the idea that emotion regulation strategies are stable individual psychological behavioral patterns, the status of mental health is the result variable formed by the interaction of the environmental variables with the individual characteristics of the person. Therefore, the current research expected that the use of emotion regulation and emotional self-efficacy would mediate the relationship between family relations and student psychological well-being at vocational colleges.

## 2 Materials and methods

### 2.1 Subjects of study

This study focused on students of a vocational and technical college in Shaanxi Province, China. The researchers employed random sampling to distribute 2,300 questionnaires in November 2022; 2,026 valid responses were collected. The effective response rate was 88.09%. The study participants included 1,081 males and 945 females. The average age was 18.92 ± 3.71 years. There were 458 children and 1,568 non-single children. This study was approved by the Ethics Committee of the Mental Health Education Center at Shaanxi Vocational and Technical College (Approval No. 000181), and written informed consent was obtained from all participants.

### 2.2 Research instruments

#### 2.2.1 Family adaptability and cohesion scale II in Chinese

In this measure (Zhang G. R. et al., 2023; Zhang L. et al., 2023), 30 items were divided into two factors: adaptation (14 items) and family cohesion (16 items). Its rating has a 5-point range using the terms *never* (1) and *always* (5). Higher scores indicated better family functioning. The original scale assesses perceived and ideal family functioning. The items were identical for the perceived and ideal sections. In this study, only perceived family functioning was measured. Cronbach’s alpha for this questionnaire was 0.963; the McDonald’s Omega coefficient for this questionnaire was 0.962.

#### 2.2.2 The questionnaire on emotion regulation

This questionnaire was used to measure the utilization of the two emotion regulation techniques. It is a 10-item questionnaire (Gross and John, 2003), with each item rated on a 7-point scale. Respondents were asked to select a number between 1 and 7, with 1 indicating *strongly agree* and 7 indicating *strongly disagree*. The higher the score of a certain strategy in the questionnaire, the higher the probability that the participant tends to use that specific strategy to regulate their emotions. The Cronbach’s alpha coefficient for this questionnaire was 0.933; the McDonald’s Omega coefficient for this questionnaire was 0.932.

#### 2.2.3 The public health emergencies questionnaire

Interestingly, this questionnaire examined aspects of mental health among the population during the COVID-19 pandemic. The questionnaire comprised 25 questions divided into five dimensions (Zhang G. R. et al., 2023; Zhang L. et al., 2023): depression, nervous fatigue, fear, obsessive-compulsive tendencies, and illness anxiety disorders. The scoring is based on four ratings: 0, representing no points; 1, representing mild points; 2, representing moderate points; and 3, representing severe points. The items in a dimension were divided by the total score to obtain the average score. The average score indicated the degree of severity of each dimension. The Cronbach’s alpha coefficient for this questionnaire was 0.963; the McDonald’s Omega coefficient for this questionnaire was 0.964.

#### 2.2.4 The regulatory emotional self-efficacy scale

This scale has been modified to fulfill domestic conditions and the strained processes of scale-responsive surgery (Dou et al., 2013). The results of the validation suggested an internal consistency coefficient of 0.788 and a split-half reliability of 0.751. According to these findings, the scale can, to some extent, be used as a psychometric instrument to assess emotional regulation self-efficacy in Chinese adolescents. The new scale consists of 17 questions and is rated on a 5-point scoring scale. The higher the score, the higher the self-efficacy. The Cronbach’s alpha coefficient for this questionnaire was 0.964; the McDonald’s Omega coefficient for this questionnaire was 0.962.

### 2.3 Study procedure

The participants were provided with anonymous questionnaires. They were guided according to standardized instructions and methods, and the questionnaires were completed uniformly after they fully understood the requirements. Responses were collected uniformly after completion.

### 2.4 Statistical methods

The collected data were entered into SPSS 20.0 software and analyzed using Spearman’s correlation analysis and descriptive statistical analysis. Mediation effects were tested using the bootstrap method.

## 3 Results

This study employed the PROCESS macro in SPSS 22.0 to examine the mediating mechanism between family relationships and psychological well-being. The primary rationale for selecting the PROCESS macro is its capacity to estimate direct and indirect effects within a regression framework simultaneously. Furthermore, it enables robust testing of mediating effects through bootstrapping, thereby circumventing the limitations of traditional causal stepwise methods concerning statistical power and assumptions of normality.

For data processing, the collected data were entered into SPSS 20.0 and analyzed using descriptive statistical and Spearman correlation analyses. The results revealed significant correlations between variables (*p* < 0.05) (see Table 1).

**Table 1:** Correlation analysis among variables.

Variable	Cognitive reappraisal	Expressive suppression	Self-efficacy in manifesting joyful feelings	Self-efficacy in handling adverse feelings	Mental health	Family relationships
Cognitive reappraisal	1	0.717**	0.667**	0.707**	−0.190**	0.593**
Expressive suppression		1	0.354**	0.566**	−0.059**	0.324**
Self-efficacy in expressing positive emotions			1	0.639**	−0.219**	0.564**
Self-efficacy in managing negative emotions				1	−0.279**	0.602**
Mental health					1	−0.273**
Family relationships						1

*** represents *p* < 0.001.

Model 4 (a simple mediation model) was used in SPSS 22.0 to mediate the relationship between family relations and mental health, utilizing the PROCESS-based macro. The variables were standardized. Family relationships were identified as independent variables, and cognitive reappraisal, expressive suppression, self-efficacy in expressing positive emotions, and self-efficacy in abstracting negative emotions were considered as examples of mediating variables; the dependent variable was mental well-being. A nonparametric percentile bootstrap test was conducted using bias-corrected data. Concerning the 95% confidence interval, the test was resampled 5,000 times.

Table 2 provides evidence that family relationships are significantly predictive of mental health (*β* = −0.273, *t* = −12.754, *p* < 0.001). According to Cohen’s (1988) classification of effect sizes, this effect may be regarded as a moderate correlation. The effect of family relationships was also significant in predicting mental health, although the direct effect was reduced after introducing the mediator variables into the analysis; the difference remained significant (*β* = −0150, *t* = −5.248, *p* < 0.001). This represents a low level of correlation. The effect of family relationships on cognitive reappraisal was strong and positive (*β* = 0.593, *t* = 33.109, *p* < 0.001). This constitutes a highly correlated level. Family relationships had a strong and negative impact on expressive suppression (*β* = −0.324, *t* = −15.393, *p* < 0.001). This represents a moderate level of correlation and a strong and positive effect of family relationships on self-efficacy in expressing positive emotions (*β* = 0.564, *t* = 30.745, *p* < 0.001). This constitutes a highly correlated level; cognitive reappraisal was not of any significant predictive value in mental health (*p* > 0.05). Expressive suppression was found to be a significant predictor of mental health, with a positive predictive value of 155 (*t* = 4.787, *p* < 0.001). Expressing positive emotions was not a perfect indicator of mental health (*p* > 0.05). There is a negative predictive association between self-efficacy in managing negative emotions and mental health (*β* = −0.249, *t* = −7.446, *p* < 0.001).

**Table 2:** Regression analysis among variables.

Outcome variable	Predictor variable	*R*	*R2*	*F*	*β*	*t*
Mental health	Family relationships	0.330	0.109	49.408***	−0.150	−5.248***
	Cognitive reappraisal				−0.029	−0.681
	Expressive suppression				0.155	4.787***
	Self-efficacy in expressing positive emotions				−0.011	−0.334
	Self-efficacy in managing negative emotions				−0.249	−7.446***
Mental health	Family relationships	0.273	0.074	162.653***	−0.273	−12.754***
Cognitive reappraisal	Family relationships	0.593	0.351	1096.215***	0.593	33.109***
Expressive suppression	Family relationships	0.324	0.105	236.946***	−0.324	−15.393***
Self-efficacy in expressing positive emotions	Family relationships	0.564	0.318	945.280***	0.564	30.745***
Self-efficacy in managing negative emotions	Family relationships	0.602	0.362	1147.972***	0.602	33.882***

*** represents *p* < 0.001.

Table 3 provides evidence that the ones mentioned above are not equal to zero in the groups of the direct relationship between family relationships and mental health, the mediation effect of expressive suppression of the direct relationship between family relationships and mental health, mediation effect of self-efficacy in handling negative emotions, and the mediation effect, as appears in the bootstrap 95% confidence intervals. The results indicate that family relations directly influence mental health, especially in combination with the mediating impact of self-efficacy on unpleasant emotions and the repression of expressions. The direct effect was 45.05% and the mediation effect accounted for 55.04% of the total effect.

**Table 3:** Decomposition of the total, direct, and mediation effects.

Path	Effect value	Bootstrap standard error	Boot 95% CI	
			Lower limit	Upper limit
Direct effect	−0.150	0.033	−0.214	−0.085
Total indirect effect	−0.123	0.025	−0.172	−0.075
Indirect effect 1	−0.017	0.027	−0.070	0.034
Indirect effect 2	0.050	0.011	0.029	0.073
Indirect effect 3	−0.006	0.021	−0.046	0.035
Indirect effect 4	−0.150	0.023	−0.196	−0.107
Total effect	−0.273	0.024	−0.319	−0.225

The indirect effects can be described as Family Relationships ≥Cognitive Reappraisal ≥Mental Health; Family Relationships ≥Expressive Suppression ≥Mental Health; Family Relationships ≥Self-Efficacy in expressing positive emotions = Mental Health; Family Relationships ≥Self-Efficacy in managing negative feelings = Mental Health.

## 4 Discussion

These findings imply that the linkage between family relationships and mental health is significant in a direct relation, and the relationship has a mediating effect involving expressive suppression and self-efficacy in processing negative effects. Under normalized prevention conditions, students with stronger relationships with their families have a lower chance of developing poor mental conditions, depression, nervous fatigue, fear, obsessive-compulsive dispositions, and disease anxiety disorders. This observation is consistent with those previously reported in this area. According to the study conducted by Yu et al. (2021), the number of suboptimal mental health symptoms was significantly negatively correlated with family cohesion and adaptability scores. Low cohesion suggests limited emotional communication among family members. This limitation leads to misunderstanding and communication barriers. Consequently, family members struggle to establish a foundation for emotional communication and support. This lack of foundation and weakened sense of responsibility may cause students to develop self-centered and indifferent personality traits. These personality traits can result in resistance and rejection during social adaptation, leading to emotions such as resistance and rejection. Ultimately, these emotions negatively affect mental health (Bonnaire and Phan, 2017). However, overly intimate and entangled family relationships have drawbacks as they may cause students to develop rigid and singular adaptation patterns. Children are often overly dependent on their families. They rarely experience social concern and trust, and lack a sense of independence. In interactions, they may become passive or isolated and often have weak resilience. Over time, mental health is negatively affected by these conditions.

This study discovered that family relationships present a strong positive predictive impact on cognitive reappraisal in emotion regulation and a negative predictive impact on expressive suppression. These results were consistent with those of earlier studies (Luo, 2013; Qiao, 2019). The formation and choice of emotion-regulation strategies are long-term processes shaped by years of interaction with family members. In healthy functioning families, cohesion is usually high, communication among family members is equal and effective, and relationships between parents and children are harmonious. Consequently, family members derive ample emotional value from these interactions. This emotional value enables them to approach problems with empathy and actively manage emotional issues. Conversely, in families with dysfunctional functioning, communication between members is scarce, emotions are distant, members may be impulsive and irritable, and they often have a mutual aversion. Consequently, they have poor resistance to and regulation of negative emotions. Family disputes are also exacerbated by problems, and as such, the family system is in a state of disarray. The impact of family relations on the efficacy of emotional regulation was observed in the data, which showed a significant positive predictive effect on self-efficacy in recounting positive emotions and self-efficacy in regulating negative ones. The impact of family relations is consistent with the findings of Xie and Fan (2021). They found in their study that the more your family functions well, the higher your regulatory emotional self-efficacy.

Mental health outcomes were predicted to have a significant positive impact on expressive suppression. This reaction shows that the expressive suppression strategy of emotion regulation increases the vulnerability of students to negative mental conditions such as depression, nervous fatigue, fear, obsessive-compulsive behavior, and illness anxiety disorder in a normalized preventive context. Recent research on expressive suppression has focused on whether expressive suppression is an appropriate emotion regulation strategy. Long-term conscious efforts to reduce emotional experiences via emotional suppression are generally ineffective. A previous study (Hofmann et al., 2009) proved that a reappraisal strategy is more effective than a suppression strategy for emotion regulation. However, this research was developed within a Western context and may not be universally applicable across different cultural backgrounds. Recent research indicates that expression suppression may be associated with positive emotions (Fernandes and Tone, 2021). In cultures characterized by collectivism and relationship orientation, higher endorsement of horizontal individualism and vertical collectivism was associated with greater use of suppression strategies (Klein et al., 2024). In cultures centered on collectivism and relationship orientation, emotional expression is often subject to strong social constraints. Maintaining interpersonal harmony and fulfilling social role responsibilities are accorded greater value. When an individual’s emotional regulation strategies align with their culture’s prevailing values, these strategies are more likely to enhance their sense of control and social efficacy, thereby exerting a positive influence on mental well-being.

Self-efficacy in coping with negative emotions is a strong negative predictor of mental health. This reveals that a higher level of self-efficacy in coping with negative emotions makes the student less susceptible to a poor state of mind, such as depression, anxious fatigue, fear, the tendency to have obsessive-compulsive disorders, and illness phobia, under normal preventive conditions. People with high emotional regulation self-efficacy can manage negative outcomes, leading to discordant negative emotions. Consequently, they can focus on remaining motivated at work and in school (Zou et al., 2023).

This means that family relationships may directly and indirectly influence mental health by mediating the effects of expressive suppression and self-efficacy on coping with negative emotions. These results suggest that good emotional management leads to increased psychological adaptation. The significant correlation between family functioning, emotion regulation, and mental health suggests that the psychological problems prevalent among college students in a normalized preventive context can be largely attributed to family factors. These problems directly reflect family relationships. However, existing family relationships are not formed overnight; rather, they develop gradually over time. Changing these inherent relationships is challenging, but students can be guided to recognize the effects of existing patterns on their current lives, master emotion management methods, and achieve a positive and healthy physical and mental state by enhancing their emotion regulation self-efficacy and adjusting their emotion regulation strategies.

The findings demonstrate a significant association between family relationships and mental health, with the direct effect remaining significant after accounting for emotion regulation strategies and emotion-related self-efficacy, indicating partial mediation. Family relationships were positively related to cognitive reappraisal and emotion-related self-efficacy and negatively related to expressive suppression; however, only expressive suppression and self-efficacy in managing negative emotions were significantly associated with mental health. These results suggest that these two factors represent key mechanisms linking family relationships and mental health. This study employs a cross-sectional research design and therefore cannot draw causal inferences. The current findings merely reflect correlations between variables and cannot rule out reverse causality or the influence of potential confounding variables. Future research may further examine its causal mechanisms through longitudinal designs or experimental methods, with particular emphasis on the mediating or moderating role of internalization of cultural norms.

## Data Availability

The datasets presented in this study can be found in online repositories. The names of the repository/repositories and accession number(s) can be found at: Figshare, https://doi.org/10.6084/m9.figshare.30125464.v1.

## References

[ref1] Balogun A. G. (2019). Work-family conflict and burnout among working mothers: the role of work-family conflict self-efficacy. Gend. Behav. 17, 14224–14236. doi: 10.10520/EJC1b24bda441

[ref2] Bonnaire C. Phan O. (2017). Relationships between parental attitudes, family functioning and internet gaming disorder in adolescents attending school. Psychiatry Res. 255, 104–110. doi: 10.1016/j.psychres.2017.05.030, 28535475

[ref3] Cohen J. (1988). Statistical power analysis for the behavioral sciences. 2nd Edn. Hillsdale, NJ: Erlbaum.

[ref4] Diener E. Wirtz D. Tov W. Kim-Prieto C. Choi D. Oishi S. . (2010). New well-being measures: short scales to assess flourishing and positive and negative feelings. Soc. Indic. Res. 97, 143–156. doi: 10.1007/s11205-009-9493-y

[ref5] Dou K. Nie Y. Wang Y. Liu Y. Li J. (2013). Adolescents’ regulatory emotional self-efficacy and subjective well-being: the mediating effect of regulatory emotional style. J. Psychol. Sci. 36, 139–144.

[ref6] Fernandes M. A. Tone E. B. (2021). A systematic review and meta-analysis of the association between expressive suppression and positive affect. Clin. Psychol. Rev. 88:102068. doi: 10.1016/j.cpr.2021.102068, 34325115

[ref7] Gao Y. Wang Y. Wang Z. Ma M. Li H. Wang J. . (2024). Family intimacy and adaptability and non-suicidal self-injury: a mediation analysis. BMC Psychiatry 24:210. doi: 10.1186/s12888-024-05642-1, 38500067 PMC10946147

[ref8] Gross J. J. (1998). The emerging field of emotion regulation: an integrative review. Rev. Gen. Psychol. 2, 271–299. doi: 10.1037/1089-2680.2.3.271

[ref9] Gross J. J. John O. P. (2003). Individual differences in two emotion regulation processes: implications for affect, relationships, and well-being. J. Pers. Soc. Psychol. 85, 348–362. doi: 10.1037/0022-3514.85.2.348, 12916575

[ref10] Gross J. J. Muñoz R. F. (1995). Emotion regulation and mental health. Clin. Psychol. Sci. Pract. 2, 151–164. doi: 10.1111/j.1468-2850.1995.tb00036.x

[ref11] Hofmann S. G. Heering S. Sawyer A. T. Asnaani A. (2009). How to handle anxiety: the effects of reappraisal, acceptance, and suppression strategies on anxious arousal. Behav. Res. Ther. 47, 389–394. doi: 10.1016/j.brat.2009.02.010, 19281966 PMC2674518

[ref12] Hu L. Tang X. (2012). College students with depression and the relationship of family cohesion and adaptability. J. Shangrao Norm. Univ. 32, 91–94, 117.

[ref13] Klein N. D. Bravo A. J. Conway C. C. Keough M. T. Pilatti A. Mezquita L. . (2024). Individualism, collectivism, and emotion regulation: a cross-cultural examination among young adults from seven countries. Curr. Psychol. 43, 26007–26018. doi: 10.1007/s12144-024-06226-8

[ref14] Kökönyei G. Kovács L. N. Szabó J. Urbán R. (2024). Emotion regulation predicts depressive symptoms in adolescents: a prospective study. J. Youth Adolesc. 53, 142–158. doi: 10.1007/s10964-023-01894-4, 37985558 PMC10761508

[ref15] Li W. Quan S. (2025). Mediating effects of resilience on regulatory emotional self-efficacy and adverse mental health outcomes among college students in China. Sci. Rep. 15:25168. doi: 10.1038/s41598-025-09260-z, 40646071 PMC12254346

[ref16] Lindert J. Arndt S. Cook N. Bain P. A. Kawachi I. (2025). Positive and negative family relationships correlate with mental health conditions-a systematic review and meta-analysis. Public Health Rev. 46:1607381. doi: 10.3389/phrs.2025.1607381, 40761410 PMC12320053

[ref17] Luo Y. (2013). Study on the relationship between family functioning, emotion regulation strategies, and prosocial behavior among high school students. Xinxiang: Henan Normal University.

[ref18] Marth S. Cook N. Bain P. Lindert J. (2022). Family factors contribute to mental health conditions–a systematic review. Eur. J. Pub. Health 32:ckac129.454. doi: 10.1093/eurpub/ckac129.454

[ref19] McCraty R. Barrios-Choplin B. Rozman D. Atkinson M. Watkins A. D. (1998). The impact of a new emotional self-management program on stress, emotions, heart rate variability, DHEA and cortisol. Integr. Physiol. Behav. Sci. 33, 151–170. doi: 10.1007/BF02688660, 9737736

[ref20] Qiao Z. (2019). The relationship between family functioning, emotion regulation strategies, and sense of alienation among junior high school students. Tianjin: Tianjin Normal University.

[ref21] Xie Q. Fan Y. (2021). Correlation analysis of junior school students’ psychological capital, test anxiety, and English academic performance: correlation analysis of family functioning, regulatory emotional self-efficacy, and interpersonal relationships among college students. Psychol. Mag. 16, 14–15.

[ref22] Younas F. Gutman L. M. (2023). Parental risk and protective factors in child maltreatment: a systematic review of the evidence. Trauma Violence Abuse 24, 3697–3714. doi: 10.1177/15248380221134634, 36448533 PMC10594837

[ref23] Yu J. Wang Y. Tang X. Wu Y. Tang X. Huang J. (2021). Impact of family cohesion and adaptability on academic burnout of Chinese college students: serial mediation of peer support and positive psychological capital. Front. Psychol. 12:767616. doi: 10.3389/fpsyg.2021.767616, 34966328 PMC8710578

[ref24] Zhang L. Chen Y. Wang L. (2023). Correlations among psychological reaction, social support, and coping style in residents in post-epidemic era. Mod. Med. Health. 39, 615–618.

[ref25] Zhang G. R. Li P. S. Jia Y. B. (2023). Relationship between family cohesion/adaptability and postpartum depressive symptoms: a single-center retrospective study. World J. Psychiatr. 13, 50–59. doi: 10.5498/wjp.v13.i2.50, 36925950 PMC10011945

[ref26] Zhang P. Zhang M. Lu J. (2010). An analysis of the results of the regulatory emotional self-efficacy scale in Chinese university students. Chin. J. Clin. Psychol. 18, 568–570.

[ref27] Zhu D. Chen Y. Li L. Dunsmore J. C. (2023). Family functioning, emotion socialization, and children’s social competence: gender-specific effects in Chinese families. J. Child Fam. Stud. 32, 257–271. doi: 10.1007/s10826-022-02480-1

[ref28] Zhu J. Zhang W. Yu C. Zhou S. Sun G. Zhen S. (2015). School climate and pathological online game use among adolescents: the moderated mediation model. Psychol. Dev. Educ. 31, 246–256. doi: 10.16187/j.cnki.issn1001-4918.2015.02.15

[ref29] Zou C. Xin Z. Li Y. Su P. (2023). Role of regulatory emotional self-efficacy and positive psychological capital in the relationship between resilience and the mental health of college students. Chin. J. Sch. Health 44, 94–98. doi: 10.16835/j.cnki.1000-9817.2023.01.021

